# Supplementary material for: Managerial thinking in neonatal care: a qualitative study of place of care decision-making for preterm babies born at 27–31 weeks gestation in England
Source: BMJ Open. 2022 Jun 27;12(6):e059428. doi: 10.1136/bmjopen-2021-059428 (PMC9237905; doi:10.1136/bmjopen-2021-059428)
Supplement: Supplementary data [file bmjopen-2021-059428supp001.pdf]

OPTI-PREM observation framework

The observer will take written notes during the observations within neonatal units. Key issues to be observed are as follows, but additional relevant information should also be recorded:

- What is the event or type of activity being observed?
- What context and setting are you in?
- How does optimal place of care feature in what you're observing?
- What role does/will it play?

- Who is involved and how is the interaction managed?
- How, when and why is optimal place of care made relevant to what you are observing?
- How is optimal place of care positioned relative to other relevant issues (e.g. unit capacity, parental experience)?

- How is the idea of optimal place of care introduced to parents, and how is it received?
- Is there broad agreement between parent and professional, or are there tensions?

- How are decisions made about optimal place of care?
- What factors influence this?
- What will happen after the episode you are observing?

- How are transfers of place of care managed?
- What factors facilitate this?
- What factors make this more difficult?

Version 1 29/08/17
IRAS 212034
