# Supplementary material for: Managerial thinking in neonatal care: a qualitative study of place of care decision-making for preterm babies born at 27–31 weeks gestation in England
Source: BMJ Open. 2022 Jun 27;12(6):e059428. doi: 10.1136/bmjopen-2021-059428 (PMC9237905; doi:10.1136/bmjopen-2021-059428)
Supplement: Supplementary data [file bmjopen-2021-059428supp002.pdf]

OPTI-PREM Interview Schedule – Staff

N.B. This topic guide will be used flexibly – the interviewer will be responsive to each individual participant and will explore related issues of interest as they arise.

Welcome and introduction

- Confirm that the interview is solely for the use of the researchers and any discussions will not be communicated in any way to anyone else.
- The report will pull together findings from all participants in the study and no individual will be identified.
- We like to audio-record each interview – is that ok?

Reiterate aims of interview

- To understand staff's views and experiences and make sure these are included in discussions about the optimal place of care for babies born between 27 and 31 weeks of gestation.
- We are interested in your views and experiences based on the type of work you do. If you do not feel you are able to comment on any area please say so.
- Do you have any questions before we start?

Background to participant

- Please could you tell me a little bit about yourself?
    - Prompt for: role, experience, seniority

Approach to decision-making on optimal place of care for these babies

- What informs decision-making on optimal place of care for these babies?
- Who is involved?
    - How/why?
- What else influences this?
    - Prompt for: baby, parents, guidelines/protocols, cot capacity
- How easy or difficult is it to decide on the optimal place of care for this type of baby?

Transfers in place of care

- How do you go about exploring or bringing about a transfer?
- What are some of the things that help with this?
- What are some of the things that make this difficult?

Relationship with parents

- How do you seek to work with parents about place of care and any transfers?
    - Why is this your approach?
    - Has it always been this way?
- Do you always manage to achieve this?
    - How/why?
- What are some of the challenges here?
- What makes this easier?

Importance of place of care

- To what extent do you think place of care is important for this group of babies?
- How much do you know about whether and how this might affect their outcomes?
- Do you have concerns about babies in sub-optimal care locations?
    - Babies in too low a level unit

    - Babies in too high a level unit

Impact on staff

- Tell me about some of the ways seeking to optimise place of care for these babies impacts on you and your work.
- Thinking particularly about decisions about and any changes in a baby's place of care, what works well for you?
    - Prompt for more detail on why/how
- What could be better?
    - Why is this a problem?
    - How could it be improved?

Anything not covered?

- Is there anything that we haven't covered in the interview that you think we should know or think about for this project?

Closing and thanks

- Check that the participant is still happy for us to use all the information provided and offer the possibility to erase sections of the recording.
- Thank for their time and contribution.
