# Supplementary material for: Managerial thinking in neonatal care: a qualitative study of place of care decision-making for preterm babies born at 27–31 weeks gestation in England
Source: BMJ Open. 2022 Jun 27;12(6):e059428. doi: 10.1136/bmjopen-2021-059428 (PMC9237905; doi:10.1136/bmjopen-2021-059428)
Supplement: Author's manuscript [file bmjopen-2021-059428.draft_revisions.pdf]

BMJ Open is committed to open peer review. As part of this commitment we make the peer review history of every article we publish publicly available.

When an article is published we post the peer reviewers' comments and the authors' responses online. We also post the versions of the paper that were used during peer review. These are the versions that the peer review comments apply to.

The versions of the paper that follow are the versions that were submitted during the peer review process. They are not the versions of record or the final published versions. They should not be cited or distributed as the published version of this manuscript.

BMJ Open is an open access journal and the full, final, typeset and author-corrected version of record of the manuscript is available on our site with no access controls, subscription charges or pay-per-view fees (http://bmjopen.bmj.com).

If you have any questions on BMJ Open's open peer review process please email info.bmjopen@bmj.com

BMJ Open

# Place of care for babies born at 27-31 weeks in England: a qualitative study of staff decision-making perspectives and practices

| | |
|---|---|
| Journal: | *BMJ Open* |
| Manuscript ID | bmjopen-2021-059428 |
| Article Type: | Original research |
| Date Submitted by the Author: | 20-Nov-2021 |
| Complete List of Authors: | Cupit, Caroline; University of Leicester, Department of Health Sciences; University of Oxford, Nuffield Department of Primary Care Health Sciences<br>Paton, Alexis; Aston University, Sociology and Policy<br>Boyle, Elaine; University Hospitals of Leicester NHS Trust, Neonatology; University of Leicester, Health Sciences<br>Pillay, Thillagavathie; Royal Wolverhampton Hospitals NHS Trust, Neonatal Unit; Dr T Pillay,<br>Armstrong, Natalie; University of Leicester, Health Sciences |
| Keywords: | NEONATOLOGY, QUALITATIVE RESEARCH, Neonatal intensive & critical care < INTENSIVE & CRITICAL CARE, HEALTH SERVICES ADMINISTRATION & MANAGEMENT, Fetal medicine < OBSTETRICS, SOCIAL MEDICINE |
| | |

SCHOLARONE™
Manuscripts

*I, the Submitting Author has the right to grant and does grant on behalf of all authors of the Work (as defined in the below author licence), an exclusive licence and/or a non-exclusive licence for contributions from authors who are: i) UK Crown employees; ii) where BMJ has agreed a CC-BY licence shall apply, and/or iii) in accordance with the terms applicable for US Federal Government officers or employees acting as part of their official duties; on a worldwide, perpetual, irrevocable, royalty-free basis to BMJ Publishing Group Ltd ("BMJ") its licensees and where the relevant Journal is co-owned by BMJ to the co-owners of the Journal, to publish the Work in this journal and any other BMJ products and to exploit all rights, as set out in our licence.*

*The Submitting Author accepts and understands that any supply made under these terms is made by BMJ to the Submitting Author unless you are acting as an employee on behalf of your employer or a postgraduate student of an affiliated institution which is paying any applicable article publishing charge ("APC") for Open Access articles. Where the Submitting Author wishes to make the Work available on an Open Access basis (and intends to pay the relevant APC), the terms of reuse of such Open Access shall be governed by a Creative Commons licence – details of these licences and which Creative Commons licence will apply to this Work are set out in our licence referred to above.*

*Other than as permitted in any relevant BMJ Author's Self Archiving Policies, I confirm this Work has not been accepted for publication elsewhere, is not being considered for publication elsewhere and does not duplicate material already published. I confirm all authors consent to publication of this Work and authorise the granting of this licence.*

For peer review only

# Place of care for babies born at 27-31 weeks in England: a qualitative study of staff decision-making perspectives and practices

Dr Caroline Cupit

Health Services Researcher

Department of Health Sciences, George Davies Centre, University of Leicester, United Kingdom. LE1 7RH

Nuffield Department of Primary Care Health Sciences, University of Oxford, United Kingdom

(Joint first author and corresponding author)

Caroline.Cupit@le.ac.uk

Dr Alexis Paton

Lecturer in Social Epidemiology and the Sociology of Health, Department of Sociology and Policy and Aston Medical School, Aston University, Birmingham, United Kingdom

(Joint first author)

Professor Elaine Boyle

Consultant Neonatologist, University Hospitals Leicester NHS Trust, Department of Neonatology

Professor of Neonatal Medicine, Department of Health Sciences, University of Leicester, United Kingdom

For peer review only



For peer review only - http://bmjopen.bmj.com/site/about/guidelines.xhtml

Professor Thillagavathie Pillay

Consultant Neonatologist, University Hospitals Leicester NHS Trust, Department of Neonatology

Professor of Neonatology, Research Institute for Health Related Sciences, Faculty of Science and Engineering, University of Wolverhampton

Professor Natalie Armstrong

Professor of Healthcare Improvement Research

Department of Health Sciences, University of Leicester, United Kingdom

Keywords: neonatology, place of care, decision-making, qualitative research

Word count: 3923



For peer review only - http://bmjopen.bmj.com/site/about/guidelines.xhtml

## Abstract:

**Objectives:** Preterm babies born between 27 and 31 weeks of gestation in England are usually born and cared for in either a Neonatal Intensive Care Unit or a Local Neonatal Unit—with such units forming part of Operational Delivery Networks. As part of a national project seeking to optimise service delivery for this group of babies (OPTI-PREM), we undertook qualitative research to better understand how staff make decisions about place of birth and care.

**Design:** Qualitative analysis of ethnographic observation data in neonatal units and semi-structured interviews with neonatal staff.

**Setting:** Six neonatal units across two neonatal networks in England. Two were Neonatal Intensive Care units and four were Local Neonatal Units.

**Participants:** Clinical staff (n=15) working in neonatal units, and people present in neonatal units during periods of observation.

**Results:** In the context of real-world neonatal practice, with multiple (and rapidly-evolving) uncertainties relating to mothers, babies, and unit/network capacity, 'best place of care' protocols were only one element of much more complex decision-making processes. Staff often made judgements from a less-than-ideal starting point, and were forced to respond to evolving clinical and organisational factors. In particular, we report that staff incorporated extensive 'managerial thinking' into their clinical decision-making about where individual babies should be born and cared for—work that was often highly time-consuming and which generated various pressures and tensions between staff.



For peer review only - http://bmjopen.bmj.com/site/about/guidelines.xhtml

**Conclusions:**

Researchers and policymakers involved in the production and application of 'best place of care' protocols for babies born at 27-31 weeks (and/or other interventions) should take account of this real-world evidence. In particular, we have drawn attention to the managerial work of balancing demand and capacity that is integrated into clinical decision-making.

For peer review only

## Strengths and limitations of this study

- This study is the first to use qualitative methods to explore practices relating to 'best place of care' using data from real-world neonatal practice.
- We undertook extensive observation (280hrs) in six neonatal care units (NICU and LNU), and captured the perspectives of a range of neonatal staff (doctors, nurses, transport specialists).
- We interviewed only a few staff from each unit, thus limiting the analytic 'depth' and comparison between sites.
- Our data did not include observations/interviews in obstetrics or maternity care, and we therefore do not discuss the operational arrangements and interdependencies between these services.

## INTRODUCTION

Babies needing medical care after birth are cared for in three types of neonatal unit across England, organised into Operational Delivery Networks: Neonatal Intensive Care Units



For peer review only - http://bmjopen.bmj.com/site/about/guidelines.xhtml

(NICU), Local Neonatal Units (LNU), and Special Care Baby Units (SCBU). Evidence suggests that outcomes are better when extremely preterm babies ($\leq$26 weeks of gestation) are born in a maternity service attached to a NICU.[1,2] For the next most vulnerable group of babies (i.e. those born between 27 and 31 weeks of gestation [hereafter referred to as born at 27-31 weeks]), there is little evidence to guide decisions about their place of birth and care. More evidence is available for babies born >31 weeks.[3] Babies born at 27-31 weeks represent around 12% of all preterm births each year in England, use twice as many neonatal bed days/year compared to extremely preterm babies, and account for over a third of all neonatal care days nationally.[4]

In the absence of clear evidence to guide optimal place of care, babies born at 27-31 weeks are born and cared for across both NICU and LNU. A national project seeking to optimise service delivery for this group of babies, OPTI-PREM,[4] is investigating whether new recommendations should be developed for subgroups of babies within the 27-31 week range, based on mortality or morbidity analyses. [Detailed criteria regarding weights and multiple babies at the lower end of the gestational age have been modified since this study was carried out.[5]] In practice, some women (who are known to be at high risk of complicated pre-term delivery) may be transferred to a specialist unit, but many others continue to deliver in a maternity service linked to a LNU. Some of these may be transferred post-delivery to a NICU due to their degree of illness or prematurity.[5] These ex-utero transfers are known to create particular stresses for babies and families[6–9] and should be avoided where possible.[10] In the past, transfers have been shown to be related to regional demand and capacity issues.[11–13] However, a high number of transfers have continued



For peer review only - http://bmjopen.bmj.com/site/about/guidelines.xhtml

despite neonatal service reorganisation.[14] There is little recent evidence on how decisions about place of care are made in practice.

This article reports a qualitative study (undertaken as part of the OPTI-PREM study) to understand how decisions about place of care are being made and operationalised for this group of babies. We are aware of no previous ethnographic work addressing place of care decision-making in this clinical context.

## METHODS

We undertook an ethnographic study in two Neonatal Operational Delivery Networks in England. Within each network, a NICU and two attached LNU were included in the study (six neonatal units in total). From January to October 2018, observations and staff interviews were conducted in order to explore decision-making about place of care in a real-world context.[15–17] We aimed to answer the question: 'What are staff perspectives and practices in relation to place of care for babies born at 27-31 weeks?' The wider study also captured experiences of parents. However, as parents were not directly involved in making decisions about babies' place of care, data from parent interviews are not presented in this article.

Sites were identified and invited to participate through the study principal investigator's professional network. Staff working in the units were introduced to the study by members of the OPTI-PREM project team. Written information on the project was provided. Observations were conducted by AP (an experienced qualitative researcher), and included



For peer review only - http://bmjopen.bmj.com/site/about/guidelines.xhtml

ward rounds, daily clinical activities, bedside discussions with parents, transfer discussions and referrals, and transfers themselves. Verbal consent for observations was sought from all staff and parents in the unit prior to each period of observation. Anonymised field notes were made. AP proactively responded to sensitive situations and, in some cases, made decisions to withdraw temporarily.

As well as undertaking informal discussions with staff and parents during ethnographic data collection, AP also conducted formal interviews with staff. All doctors and nurses working at the units on observation days were approached for interview. Participation was voluntary. Fifteen staff agreed to be formally interviewed (many more contributed to the ethnographic element of the study, through being observed and via informal chats) and written consent was obtained. Most non-participation was due to challenges with scheduling around shifts. Interviews were semi-structured based on a topic guide developed through literature review and discussions within the project team. Interviews were conducted by AP on site, lasted up to an hour and were audio-recorded, transcribed verbatim, and de-identified.

Analysis was inductive and interpretative, with AP using the constant comparative method to undertake initial coding.[18] CC also carried out detailed reading of transcripts/field notes, and employed an analytic approach known as 'institutional ethnography'[19–22] to study place of care decision-making as part of socially-organised work processes.[23–26] NVivo software was used to organise and retrieve data.



For peer review only - http://bmjopen.bmj.com/site/about/guidelines.xhtml

**Patient and Public Involvement**

The OPTI-PREM project was supported by a Bliss[27] volunteer parent panel, which was involved in designing and overseeing the research.

## RESULTS

In total, 280 hours of observations were conducted and 15 members of staff were interviewed across the six units. A breakdown of professional roles is shown in Table 1.

| Site | Designation | Clinician Type | Number of participants (Total=15) |
|---|---|---|---|
| Site 1 | NICU | Doctor (Consultant) | 1 |
| Site 2 | NICU | Doctor<br>Nurse | 1<br>1 |
| Site 3 | LNU | Doctor (Registrar)<br>Nurse | 1<br>2 |
| Site 4 | LNU | Doctor (Consultant)<br>Nurse | 1<br>1 |
| Site 5 | LNU | Doctor (Consultant) | 1 |
| Site 6 | LNU | Doctor (Consultant)<br>Nurse | 1<br>2 |
| Working across sites | | Transport Nurse | 3 |

Table 1: Participant overview

The findings reported here are presented in two main themes. The first theme highlights the complex, multi-factorial context in which staff made decisions about place of care, and consequently the difficulties of producing and applying 'best place of care' protocols, even for *subgroups* of babies within the 27-31 week range. The second theme specifically focuses



For peer review only - http://bmjopen.bmj.com/site/about/guidelines.xhtml

on how staff integrated ‘managerial thinking’ about demand and capacity management (within the network’s contractual framework) into clinical decision-making about the ‘best place of care’ for each individual baby.

**‘Best place of care’ protocols within contextualised decision-making processes**

Place of birth and care was to a large extent governed by regional protocols, which determined what should happen in the case of an expected preterm birth at 27-31 weeks gestation.[5,28,29] For example, regional care pathways meant that LNU should only accept and care for babies born at ≥27/28 weeks, depending on the unit. For expectant preterm births in earlier gestational subgroups, the woman (with baby in utero) should be transferred to a maternity service attached to a NICU, assuming mother and fetus were considered adequately stable. Otherwise, the baby could be transferred post-delivery to a NICU for ongoing care if needed. Neonatal staff looked to such protocols (along with dynamic assessment of unit/network resources) to determine how to proceed.

> *Majority of the time, it’s quite a simple decision, for example guidelines we can't deliver, or we shouldn’t manage anyone less than 27 weeks here […] And [we] have clear guidelines of how many nurses we need to run an ITU [intensive therapy unit], how much space you need and all that.*
>
> *Doctor12, LNU*

Although, as indicated above, these decisions were often simple *in principle*, they involved *in practice* multiple uncertainties relating to both mother and baby’s condition. Staff knew that, in an idealised scenario, the safest possible option would be for all mothers to have



For peer review only - http://bmjopen.bmj.com/site/about/guidelines.xhtml

access to the specialist resources of a maternity service linked to a NICU, but they also recognised that this level of specialist care was unnecessary for some babies. The problem was that, although some information was known prior to delivery (and an informed guess made about a baby's subsequent condition), a baby's post-delivery condition was unpredictable:

> *These are very notorious group because they can behave like an extreme premature or they can just need a bit of support […]. It's very difficult to predict these babies.*
>
> *Doctor12, LNU*

Pre-delivery, it was difficult to predict which babies could be safely managed on LNU, and decisions were reliant on local expertise. Staff considered this critical decision-making expertise to be lacking in some LNU:

> *We can scan but we don't have the expertise to confidently say there's no bleeds going on or the head's normal. So just small things that add up to not having good care or optimal care for the extreme premature baby just because we are not funded, we've not got the personnel or the skills because we don't deal with that so often.*
>
> *Doctor12, LNU*

Staff therefore considered that there was extra risk attached to delivering this group of babies in many LNU [expertise and equipment varied from unit to unit].



For peer review only - http://bmjopen.bmj.com/site/about/guidelines.xhtml

Not only were babies unpredictable, but so too were mothers and families. For example, a mother might not travel to the preferred NICU due to imminent labour, or social circumstances. Units therefore had to handle imminent deliveries regardless of whether or not they were equipped to care for the baby according to recognised standards and guidelines for neonatal care. Even mothers classified in advance as being 'high risk' (and therefore booked at a NICU) often presented in labour at their local unit, which was then obliged to manage the delivery:

> *Triplets were booked for Site 1 but were born at Site 5. Consultant said this happens a lot; that high risk pregnancies are booked at Site 1 but then Mum presents at Site 5 too far along to be moved.*
>
> *Observation, Site 5, LNU*

Often, a suboptimal delivery at an LNU was not due to the unpredictability of mother or baby, but due to capacity issues in local NICU or lack of availability of transport teams (that were needed to safely carry out some in-utero transfers). Units often ended up accepting babies for which they did not have appropriate capacity (e.g. equipment/staffing):

> *[The nurse said:] Sometimes [the LNU] is full and [they] tell people to rock up [here] without checking [we] can take the pressure.*
>
> *Observation, Site 5, LNU*

A unit's own capacity management had to be undertaken in relation to capacity issues across the network/region. As the excerpt above indicates, units were not always aware



For peer review only - http://bmjopen.bmj.com/site/about/guidelines.xhtml

of/responsive to other units' management concerns and this was exacerbated by variation in policies and fluctuating capacity across units of the same designation:

> *[…] every hospital is different. Policies are different. Maybe it will be in time standardised within the NHS*
>
> *Nurse10, NICU*

As highlighted above, many of the 27-31 week babies in this study were born in LNU—and this included those who did not meet established local criteria. [See Edwards and Impey[30] who found that, even in the <27 week group of extremely preterm babies, only 50% were actually born in a specialist unit, despite this being the recommendation.] In practice, protocols were frequently contravened due to limited capacity across obstetrics/maternity services and NICU, along with the other factors relating to mother and baby that we have already outlined. Other babies were born in NICU (e.g. due to the mother's home being in close proximity) even though they could be adequately cared for on an LNU.

In practice, therefore, many place of care decisions were not taken from a stable, pre-clinical position, but within an existing clinical context (either LNU or NICU)—in which care for mother and baby was already underway and rapidly evolving:

> *If the birth is imminent then we just go for it. Personally I've dealt with babies who are twenty-three-and-a-half weeks onwards [at another unit]. So personally speaking I'm comfortable and I'm pretty sure my nursing colleagues*



For peer review only - http://bmjopen.bmj.com/site/about/guidelines.xhtml

*are pretty comfortable. So we deal with them, but as soon as the baby comes out, one of my colleagues starts looking around hunting for a bed.*

*Doctor20, LNU*

In such situations, staff had to cope with whatever happened, and prepare to transfer the baby (if required) after delivery.

In other circumstances, staff made calculated judgements to keep mothers at a local hospital, based on their experience and dynamic knowledge of multiple contextual factors:

*On a December cold, wintery night and a mum came in-utero [with triplets]. And there was possibly a small window for her to go elsewhere, but I carefully calculated the risk and said "your twenty-nine-weekers have very good growth for all those babies and, it's winter and there is a chance that things might happen en route...". At the time I had a very experienced registrar with me and I did have another member of the team who could definitely come back [into work after leaving shift]. And this mum had had steroids as well in the last week. So looking at all those calculations I actually did look after them.*

*Doctor30, LNU*

Assessment of the best place of care was often re-evaluated following delivery, with some decisions being relatively straightforward (in principle at least)—based on urgent clinical needs—whilst others required more complex assessment of the pros and cons within the wider unit/network context.



For peer review only - http://bmjopen.bmj.com/site/about/guidelines.xhtml

When staff made decisions (with baby either in-utero or ex-utero), they not only applied protocols but also attended to a huge range of uncertainties and a rapidly-evolving situation relating to mother, baby, and also unit/network capacity, which affected how those protocols were applied.

**'Thinking managerially' about where individual babies should be born and cared for**

Decisions about place of care for most of the babies born at 27-31 weeks was highly contextualised and involved assessment of the particular needs of individual babies. It was clear that all staff aspired to the best quality care for individual babies. However, each individual baby's clinical assessment was made within a socially-organised clinical context. Our data show that resources (and their distribution across networks) significantly shaped place of care decisions about individual babies born at 27-31 weeks—with specialist maternity services and NICU only being available in city locations, and without the necessary capacity to accommodate all 27-31 week births.

More specifically, we found that a large amount of staff work was dedicated to matching the (either anticipated or known) clinical needs of the neonatal baby population ('demand') with the availability of expertise and technologies ('capacity')—both of which were scattered geographically across a region, and were constantly being reconfigured. Staff drew not only on knowledge of the baby's needs (e.g. requirement for ventilation, tube feeding, surgery) but also on knowledge of capacity and resources within their own unit within the context of the wider network:



For peer review only - http://bmjopen.bmj.com/site/about/guidelines.xhtml

*We like to think managerially as well [as clinically]. If [another unit with limited specialist capacity] has a twenty-eight week [baby] and they ask whether we would like to accept, we are more than happy. Because, not only it's good for our practice that we keep on getting prem babies, it [also] brings money to the [hospital]. So we are always looking for kids...*

*Doctor20, LNU*

Staff spoke of their 'network responsibility' to try to accommodate babies—a responsibility linked to national tariffs for different categories of care[31,32] and locally negotiated contracts and payments.[33] They knew that, in relation to this 'responsibility', they needed to "think managerially" about capacity and resources. Such thinking (and the associated decision-making) was related to the unit's need to maximise income, whilst avoiding 'unsafe' practice (as determined by capacity/staffing protocols[10]). In the excerpt above, one unit was short of the required capacity, whilst another was looking to ensure that available capacity was generating income.

Staff were locked into continuous work to review their own capacity and respond to the requests of other units within the network. 'Capacity' in this context included many different elements—including staff mix, qualifications, experience, space, and technologies:

*So in our [LNU], [we can take] twenty eight weeks and above. And then we look at ourselves. How are we? Is there enough nursing staff? Are there enough doctors? And then we look at bed capacity, and [intensive care capacity], whether there's enough space. [...] Sometimes it happens that we shift babies out because of capacity.*



For peer review only - http://bmjopen.bmj.com/site/about/guidelines.xhtml

*Doctor20, LNU*

All units logged requests for transfers from other units, noting why they accepted or refused. One transport nurse emphasised that units did their best to accommodate babies who were vulnerable to deterioration as a consequence of transfer:

*"[Units] try and shuffle to make [babies] fit in."*

*TransportNurse05A*

However, in all six units, staff or cot capacity were the most often cited reasons for a refusal.

Units were paid for their 'activity' (number of babies), so accepting new babies was important for income generation as well as to maintain the unit's status (also linked to the category of babies they were allowed to accept):

*You have to accept, because we are an LNU, we cannot afford our activity to go down. Because [then] we'll be relegated to a [non-specialist] unit. So we won't want to be seen as refusing […] We can get de-skilled […] and the more activity we do, the more funds we get [so] it's a win-win situation.*

*Doctor26, LNU*

LNU therefore tried to retrieve babies who had been transferred to NICU, as soon as they were clinically ready for repatriation:

*There is a baby here from an out-of-network hospital. [The hospital] has been ringing to see if it can come back.*

*Observation, Site 1, NICU*



For peer review only - http://bmjopen.bmj.com/site/about/guidelines.xhtml

For peer review only

In addition, each baby attracted a different tariff, according to the category of care that was required[31,32]—and this was an important consideration:

> *While waiting, [another hospital] has rung up with an ex-utero transfer (twins). "We must be the only place with beds!" Everyone is excited to take the babies as it is an ITU transfer and that brings in a lot of money. [Staff member] singing "one thousand and twenty-one pounds. Thank you!"*
>
> *Observation, Site 3, LNU*

However, maximising capacity, and meeting the needs of very sick babies, was sometimes in tension with providing a broad spectrum of neonatal delivery for the local population. For example, babies who were clinically stable, and whose local unit was a NICU, were often transferred out, sometimes to an inconvenient location, in order to accommodate babies with more complex needs:

> *The units are very good; if it is a really sick baby that's got a specific problem then they're very good at trying to make beds, so often we'll move other babies out … we do jiggling around quite a lot.*
>
> *TransportNurse05*
>
> *So it's finding a divide between running a hospital (just purely thinking about the service and how much activity you can churn through at the high end of intensive care) versus running a service that makes sure you provide the right service for the families of your local population*
>
> *Doctor06, NICU*



For peer review only - http://bmjopen.bmj.com/site/about/guidelines.xhtml

Unlike decisions that were made based purely on clinical need, decisions orientated entirely to demand and capacity management took an emotional toll on staff:

> *I think the hardest conversation is the move out for capacity reasons. So making a decision to impact significantly on a family […] for the care of a different baby who they don't have nothing to do with can be quite difficult sometimes.*
>
> *Doctor09, NICU*

Capacity-related issues could also create tension between staff groups:

> *The doctor was complaining about how there is no standardised practice for closing the unit […] The nurses always want to operate at a gold standard, but it is impossible to operate at that level because the unit is always falling short due to staff shortages.*
>
> *Observation, Site 1, NICU*

Senior staff were drawn into extensive work to account for capacity-related decisions, and they passed this pressure onto staff within their units:

> *Consultants want to have a better way of recording why babies can't be accepted [for upwards reporting]. They want specifics, so they were saying things like "Why have you said we're busy? What is it about the category of babies in today that means we can't take another one?"*
>
> *Observation, Site 1, NICU*



For peer review only - http://bmjopen.bmj.com/site/about/guidelines.xhtml

Our findings highlight that clinical decisions about the ‘best place of care’ for individual babies were, in practice, and to a significant extent, orientated to demand and capacity management, as part of the unit’s contractual arrangements within the network. In practice, ‘clinical’ and ‘managerial’ ways of thinking were entirely intertwined. In some situations, these different strands of knowing about what was ‘best’ aligned well (and appeared to present no difficulties for staff, parents, or babies). In others, they were in tension. Overall, a large amount of staffing resource was involved in negotiating babies’ individual needs and units’ resources. Staff spent considerable energies working to meet the clinical needs of each individual baby, whilst also balancing the needs of other babies (whether they were being transferred or staying put in their birth hospital)—within the constantly-reconfiguring distribution of resources within the network.

## DISCUSSSION

This paper provides empirical evidence of how ‘best place of care’ protocols are incorporated into practical ‘real world’ decision-making for babies born between 27-31 weeks of gestation in England.

Our findings are highly relevant to stakeholders who see solutions in standardised protocols for neonatal place of care. We found that such protocols were only one element of much more complex decision-making processes. This has implications for the production and application of evidence relating to ‘best place of care’. In relation to *production* of evidence, studies attempting to assess the clinical outcomes of babies born in NICU compared with those born on LNU are likely to be challenging as NICU and LNU vary considerably within



For peer review only - http://bmjopen.bmj.com/site/about/guidelines.xhtml

these classifications, and the characteristics of babies born in each are not standardised. Transfers between units are also likely to make comparisons extremely difficult. In relation to *application* of evidence, standardised protocols for this group of babies may also present difficulties within a context that involves un-standardised babies, mothers, geographies, resourcing and so on. Even if future evidence indicates that some subgroups of babies in the 27-31 week range are (theoretically) best to be born and/or cared for in hospitals with a NICU, such highly specialist and costly facilities will not be on the doorstep of all mothers, and will undoubtedly have limited capacity. Stakeholders should be alert to the situatedness of decision-making and the potential for unintended consequences following the introduction of new protocols.[34]

Importantly, we have drawn particular attention to the managerial work (and 'managerial thinking') that is involved in staff decision-making about place of care. When staff make decisions about place of care for an individual baby, they integrate clinical knowledge about that baby's needs with management understandings of what is 'best'. We have highlighted that neonatal staff were highly skilled at making complex judgements about place of care based on their in-situ knowledge about the needs of babies, and the resources (e.g. staffing, equipment, cots) that were available to meet those needs. Although sometimes it appeared that 'clinical' and 'managerial' ways of knowing were aligned, at other times they came into conflict. Institutional systems and processes that organised neonatal care (contractual funding arrangements, safe staffing protocols etc.) often created difficulties as they coordinated neonatal work. We have highlighted that staff spent a huge amount of time reviewing and managing their unit's capacity—work that inevitably diverted them from



For peer review only - http://bmjopen.bmj.com/site/about/guidelines.xhtml

other activities. Management of capacity and funding flows also put pressure on staff, impacting on morale as well as their time to support babies and their families. It is important for stakeholders to understand how contractual and funding configurations shape clinical decision-making in practice.[35] Knowledge of what happens in real-world neonatal practice can help ensure that new policies, protocols, or other interventions are developed in light of contextual challenges and thus deliver intended improvements in care.

## CONCLUSIONS

Researchers and policymakers considering the 'best place of care' for babies born at 27-31 weeks (or other interventions) should take account of this real-world evidence. In particular, we have drawn attention to the managerial work involved in balancing demand and capacity that is integrated into clinical decision-making.

## Acknowledgements

The authors thank all the staff whose experiences are reported in this article, and the other participants involved in the OPTI-PREM study. Natalie Armstrong is supported by a Health Foundation Improvement Science Fellowship and also by the National Institute for Health Research (NIHR) Applied Research Collaboration East Midlands (ARC EM). The views expressed are those of the authors and not necessarily those of the NHS, the NIHR or the Department of Health and Social Care.



For peer review only - http://bmjopen.bmj.com/site/about/guidelines.xhtml

## Author contributions

Obtained funding: TP, EB, NA. Study design: NA. Data analysis: CC, AP. Drafting manuscript: CC, AP. Interpretation to neonatal practice: TP, EB. Conceptualisation and critical revision of the manuscript: CC. All authors approved the final version.

CC and AP contributed equally to this paper.

## Funding

This work was supported by the National Institute for Health Research, Health Services and Delivery Research Stream, Project number 15/70/104.

## Competing interests

None declared.

## Disclaimer

The views expressed in this article are those of the authors and not necessarily those of the NHS or NIHR.

## Patient consent for publication

Not required

## Ethics approval

The study was approved by the North East – Tyne and Wear South REC (IRAS 212304) and is registered with Clinicaltrials.gov (NCT02994849).

## Provenance and peer review

Not commissioned. Externally peer-reviewed.



For peer review only - http://bmjopen.bmj.com/site/about/guidelines.xhtml

For peer review only

## Data availability statement

No data are available. The consent for this study restricts use of transcripts to the study team for confidentiality reasons. Raw data are therefore not available.

## References

1. Watson SI, Arulampalam W, Petrou S, Marlow N, Morgan AS, Draper ES, et al. The effects of designation and volume of neonatal care on mortality and morbidity outcomes of very preterm infants in England: retrospective population-based cohort study. BMJ Open. 2014 Jul 1;4(7):e004856.

2. Helenius K, Longford N, Lehtonen L, Modi N, Gale C. Association of early postnatal transfer and birth outside a tertiary hospital with mortality and severe brain injury in extremely preterm infants: observational cohort study with propensity score matching. BMJ. 2019 Oct 16;367:l5678.

3. Boyle EM, Johnson S, Manktelow B, Seaton SE, Draper ES, Smith LK, et al. Neonatal outcomes and delivery of care for infants born late preterm or moderately preterm: a prospective population-based study. Arch Dis Child - Fetal Neonatal Ed. 2015 Nov 1;100(6):F479–85.

4. Pillay T, Modi N, Rivero-Arias O, Manktelow B, Seaton SE, Armstrong N, et al. Optimising neonatal service provision for preterm babies born between 27 and 31 weeks gestation in England (OPTI-PREM), using national data, qualitative research and economic analysis: a study protocol. BMJ Open [Internet]. 2019 Aug 1 [cited 2019 Nov 25];9(8). Available from: https://bmjopen.bmj.com/content/9/8/e029421

5. NHS England. Implementing the Recommendations of the Neonatal Critical Care Transformation Review [Internet]. 2019 [cited 2021 Oct 28]. Available from: https://www.england.nhs.uk/publication/implementing-the-recommendations-of-the-neonatal-critical-care-transformation-review/

6. Sommer CM, Cook CM. Disrupted bonds–parental perceptions of regionalised transfer of very preterm infants: a small-scale study. Contemp Nurse. 2015;50(2–3):256–66.

7. Ballantyne M, Orava T, Bernardo S, McPherson AC, Church P, Fehlings D. Parents' early healthcare transition experiences with preterm and acutely ill infants: a scoping review. Child Care Health Dev. 2017;43(6):783–96.

8. Aagaard H, Hall EOC, Ludvigsen MS, Uhrenfeldt L, Fegran L. Parents' experiences of neonatal transfer. A meta-study of qualitative research 2000–2017. Nurs Inq. 2018;25(3):e12231.



For peer review only - http://bmjopen.bmj.com/site/about/guidelines.xhtml

9. Ringham CL, MacKinnon K. Mothering Work and Perinatal Transfer: An Institutional Ethnographic Investigation. Can J Nurs Res. 2019;0844562119884388.

10. British Association of Perinatal Medicine. Optimal Arrangements for Neonatal Intensive Care Units in the UK including guidance on their Medical Staffing A Framework for Practice [Internet]. London; 2021 [cited 2021 Oct 25]. Available from: https://www.swneonatalnetwork.co.uk/media/182167/bapm_optimal_arrangement_for_nicus_revision_21-4-21.pdf

11. Fenton AC, Leslie A, Skeoch CH. Optimising neonatal transfer. Arch Dis Child - Fetal Neonatal Ed. 2004 May 1;89(3):F215–9.

12. Gill AB, Bottomley L, Chatfield S, Wood C. Perinatal transport: problems in neonatal intensive care capacity. Arch Dis Child - Fetal Neonatal Ed. 2004 May 1;89(3):F220–3.

13. Fowlie PW, Booth P, Skeoch CH. Moving the preterm infant. BMJ. 2004 Oct 14;329(7471):904–6.

14. Gale C, Santhakumaran S, Nagarajan S, Statnikov Y, Modi N. Impact of managed clinical networks on NHS specialist neonatal services in England: population based study. BMJ. 2012 Apr 3;344:e2105.

15. Savage J. Ethnography and health care. BMJ. 2000 Dec 2;321(7273):1400–2.

16. Dixon-Woods M. What can ethnography do for quality and safety in health care? Qual Saf Health Care. 2003;12(5):326–7.

17. Leslie M, Paradis E, Gropper MA, Reeves S, Kitto S. Applying ethnography to the study of context in healthcare quality and safety. BMJ Qual Saf. 2014 Feb 1;23(2):99–105.

18. Glaser BG, Strauss AL. The Discovery of Grounded Theory: Strategies for Qualitative Research. Aldine Publishing Company; 1967. 292 p.

19. Smith DE. The Everyday World as Problematic: A Feminist Sociology. University of Toronto Press; 1987. 258 p.

20. Campbell M, Gregor FM. Mapping Social Relations: A Primer in Doing Institutional Ethnography. University of Toronto Press; 2002. 148 p.

21. Smith DE. Institutional Ethnography as Practice. Rowman & Littlefield; 2006. 280 p.

22. Rankin J. Conducting Analysis in Institutional Ethnography: Guidance and Cautions. Int J Qual Methods. 2017 Dec 1;16(1):1609406917734472.

23. Sinding C, Hudak P, Wiernikowski J, Aronson J, Miller P, Gould J, et al. “I like to be an informed person but…” negotiating responsibility for treatment decisions in cancer care. Soc Sci Med. 2010 Sep;71(6):1094–101.



For peer review only - http://bmjopen.bmj.com/site/about/guidelines.xhtml

24. Campbell ML, Rankin JM. Nurses and electronic health records in a Canadian hospital: examining the social organisation and programmed use of digitised nursing knowledge. Sociol Health Illn. 2016 Oct 1;39(3):365–79.

25. Reid L, Kydd A, Slade B. An inquiry into what organised difficult advance care planning conversations in a Scottish residential care home using institutional ethnography. J Res Nurs. 2018 May 1;23(2–3):220–36.

26. Cupit C, Rankin J, Armstrong N, Martin GP. Overruling uncertainty about preventative medications: the social organisation of healthcare professionals' knowledge and practices. Sociol Health Illn. 2020;42(S1):114–29.

27. Bliss. What we do [Internet]. 2020 [cited 2020 Mar 16]. Available from: https://www.bliss.org.uk/about-us/what-we-do

28. British Association for Perinatal Medicine. Optimal Arrangements for Neonatal Intensive Care Units in the UK [Internet]. British Association for Perinatal Medicine; 2014 [cited 2019 Nov 25]. Available from: https://www.bapm.org/resources/31-optimal-arrangements-for-neonatal-intensive-care-units-in-the-uk-2014

29. British Association of Perinatal Medicine. Neonatal Service Quality Indicators: Standards relating to structures and processes [Internet]. British Association of Perinatal Medicine; 2017 Jun [cited 2019 Nov 25]. Available from: https://www.bapm.org/resources/11-neonatal-service-quality-indicators-standards-relating-to-structures-and-processes-2017

30. Edwards K, Impey L. Extreme preterm birth in the right place: a quality improvement project. Arch Dis Child - Fetal Neonatal Ed [Internet]. 2019 Nov 12 [cited 2020 Mar 4]; Available from: https://fn.bmj.com/content/early/2019/11/12/archdischild-2019-317741

31. BAPM. Categories of care 2011 [Internet]. 2011. Available from: https://www.sort.nhs.uk/Media/SONeT/Guidelines/Nationalpolicies/BAPM%202011%20categories%20of%20care.pdf

32. NHS England. National Programmes of Care and Clinical Reference Groups: Neonatal Critical Care Service Specification [Internet]. 2016 [cited 2020 Feb 4]. Report No.: E08/S/a. Available from: https://www.england.nhs.uk/commissioning/spec-services/npc-crg/group-e/e08/

33. NHS England. Implementing the Recommendations of the Neonatal Critical Care Transformation Review [Internet]. 2016 [cited 2020 Feb 4]. Available from: https://www.england.nhs.uk/publication/implementing-the-recommendations-of-the-neonatal-critical-care-transformation-review/

34. Timmermans S, Epstein S. A world of standards but not a standard world: toward a sociology of standards and standardization. Annu Rev Sociol. 2010;36:69–89.



For peer review only - http://bmjopen.bmj.com/site/about/guidelines.xhtml

35. Griffith AI, Smith DE, editors. Under New Public Management: Institutional Ethnographies of Changing Front-line Work. Toronto: University of Toronto Press; 2014.

For peer review only



For peer review only - http://bmjopen.bmj.com/site/about/guidelines.xhtml

## Consolidated criteria for reporting qualitative studies (COREQ): 32-item checklist

Please indicate in which section each item has been reported in your manuscript. If you do not feel an item applies to your manuscript, please enter N/A.

For further information about the COREQ guidelines, please see Tong *et al.*, 2017: https://doi.org/10.1093/intqhc/mzm042

| No. | Item | Description | Section # |
|---|---|---|---|
| **Domain 1: Research team and reflexivity** | | | |
| Personal characteristics | | | |
| *1.* | Interviewer/facilitator | Which author/s conducted the interview or focus group? | |
| *2.* | Credentials | What were the researcher's credentials? *E.g. PhD, MD* | |
| *3.* | Occupation | What was their occupation at the time of the study? | |
| *4.* | Gender | Was the researcher male or female? | |
| *5.* | Experience and training | What experience or training did the researcher have? | |
| Relationship with participants | | | |
| *6.* | Relationship established | Was a relationship established prior to study commencement? | |
| *7.* | Participant knowledge of the interviewer | What did the participants know about the researcher? *E.g. Personal goals, reasons for doing the research* | |
| *8.* | Interviewer characteristics | What characteristics were reported about the interviewer/facilitator? *E.g. Bias, assumptions, reasons and interests in the research topic* | |
| **Domain 2: Study design** | | | |
| Theoretical framework | | | |
| *9.* | Methodological orientation and theory | What methodological orientation was stated to underpin the study? *E.g. grounded theory, discourse analysis, ethnography, phenomenology, content analysis* | |
| Participant selection | | | |
| *10.* | Sampling | How were participants selected? *E.g. purposive, convenience, consecutive, snowball* | |
| *11.* | Method of approach | How were participants approached? *E.g. face-to-face, telephone, mail, email* | |
| *12.* | Sample size | How many participants were in the study? | |
| *13.* | Non-participation | How many people refused to participate or dropped out? What were the reasons for this? | |
| Setting | | | |
| *14.* | Setting of data collection | Where was the data collected? *E.g. home, clinic, workplace* | |
| *15.* | Presence of non-participants | Was anyone else present besides the participants and researchers? | |

| | | | |
|---|---|---|---|
| *16.* | Description of sample | What are the important characteristics of the sample? *E.g. demographic data, date* | |
| Data collection | | | |
| *17.* | Interview guide | Were questions, prompts, guides provided by the authors? Was it pilot tested? | |
| *18.* | Repeat interviews | Were repeat interviews carried out? If yes, how many? | |
| *19.* | Audio/visual recording | Did the research use audio or visual recording to collect the data? | |
| *20.* | Field notes | Were field notes made during and/or after the interview or focus group? | |
| *21.* | Duration | What was the duration of the interviews or focus group? | |
| *22.* | Data saturation | Was data saturation discussed? | |
| *23.* | Transcripts returned | Were transcripts returned to participants for comment and/or correction? | |
| **Domain 3: analysis and findings** | | | |
| Data analysis | | | |
| *24.* | Number of data coders | How many data coders coded the data? | |
| *25.* | Description of the coding tree | Did authors provide a description of the coding tree? | |
| *26.* | Derivation of themes | Were themes identified in advance or derived from the data? | |
| *27.* | Software | What software, if applicable, was used to manage the data? | |
| *28.* | Participant checking | Did participants provide feedback on the findings? | |
| Reporting | | | |
| *29.* | Quotations presented | Were participant quotations presented to illustrate the themes / findings? Was each quotation identified? *E.g. Participant number* | |
| *30.* | Data and findings consistent | Was there consistency between the data presented and the findings? | |
| *31.* | Clarity of major themes | Were major themes clearly presented in the findings? | |
| *32.* | Clarity of minor themes | Is there a description of diverse cases or discussion of minor themes? | |

**When submitting your manuscript via the online submission form, please upload the completed checklist as a Figure/supplementary file.**

**If you would like this checklist to be included alongside your article, we ask that you upload the completed checklist to an online repository and include the guideline type, name of the repository, DOI and license in the *Data availability* section of your manuscript.**

Developed from: Allison Tong, Peter Sainsbury, Jonathan Craig, Consolidated criteria for reporting qualitative research (COREQ): a 32-item checklist for interviews and focus groups, International Journal for Quality in Health Care, Volume 19, Issue 6, December 2007, Pages 349–357, https://doi.org/10.1093/intqhc/mzm042

For peer review only

BMJ Open

# Managerial thinking in neonatal care: a qualitative study of place of care decision-making for preterm babies born at 27-31 weeks gestation in England

| | |
|---|---|
| Journal: | *BMJ Open* |
| Manuscript ID | bmjopen-2021-059428.R1 |
| Article Type: | Original research |
| Date Submitted by the Author: | 02-May-2022 |
| Complete List of Authors: | Cupit, Caroline; University of Leicester, Department of Health Sciences; University of Oxford, Nuffield Department of Primary Care Health Sciences<br>Paton, Alexis; Aston University, Sociology and Policy<br>Boyle, Elaine; University Hospitals of Leicester NHS Trust, Neonatology; University of Leicester, Health Sciences<br>Pillay, Thillagavathie; Royal Wolverhampton Hospitals NHS Trust, Neonatal Unit; Dr T Pillay,<br>Armstrong, Natalie; University of Leicester, Health Sciences |
| <b>Primary Subject Heading</b>: | Sociology |
| Secondary Subject Heading: | Paediatrics, Health services research, Intensive care, Medical management, Qualitative research |
| Keywords: | NEONATOLOGY, QUALITATIVE RESEARCH, Neonatal intensive & critical care < INTENSIVE & CRITICAL CARE, HEALTH SERVICES ADMINISTRATION & MANAGEMENT, Fetal medicine < OBSTETRICS, SOCIAL MEDICINE |
| | |

SCHOLARONE™
Manuscripts

*I, the Submitting Author has the right to grant and does grant on behalf of all authors of the Work (as defined in the below author licence), an exclusive licence and/or a non-exclusive licence for contributions from authors who are: i) UK Crown employees; ii) where BMJ has agreed a CC-BY licence shall apply, and/or iii) in accordance with the terms applicable for US Federal Government officers or employees acting as part of their official duties; on a worldwide, perpetual, irrevocable, royalty-free basis to BMJ Publishing Group Ltd ("BMJ") its licensees and where the relevant Journal is co-owned by BMJ to the co-owners of the Journal, to publish the Work in this journal and any other BMJ products and to exploit all rights, as set out in our licence.*

*The Submitting Author accepts and understands that any supply made under these terms is made by BMJ to the Submitting Author unless you are acting as an employee on behalf of your employer or a postgraduate student of an affiliated institution which is paying any applicable article publishing charge ("APC") for Open Access articles. Where the Submitting Author wishes to make the Work available on an Open Access basis (and intends to pay the relevant APC), the terms of reuse of such Open Access shall be governed by a Creative Commons licence – details of these licences and which Creative Commons licence will apply to this Work are set out in our licence referred to above.*

*Other than as permitted in any relevant BMJ Author's Self Archiving Policies, I confirm this Work has not been accepted for publication elsewhere, is not being considered for publication elsewhere and does not duplicate material already published. I confirm all authors consent to publication of this Work and authorise the granting of this licence.*

For peer review only

# Managerial thinking in neonatal care: a qualitative study of place of care decision-making for preterm babies born at 27-31 weeks gestation in England

Dr Caroline Cupit

Health Services Researcher

Department of Health Sciences, George Davies Centre, University of Leicester, United Kingdom. LE1 7RH

Nuffield Department of Primary Care Health Sciences, University of Oxford, United Kingdom

(Joint first author and corresponding author)

Caroline.Cupit@le.ac.uk

Dr Alexis Paton

Lecturer in Social Epidemiology and the Sociology of Health, Department of Sociology and Policy and Aston Medical School, Aston University, Birmingham, United Kingdom

(Joint first author)

Professor Elaine Boyle

Consultant Neonatologist, University Hospitals Leicester NHS Trust, Department of Neonatology

Professor of Neonatal Medicine, Department of Health Sciences, University of Leicester, United Kingdom

For peer review only



For peer review only - http://bmjopen.bmj.com/site/about/guidelines.xhtml

Professor Thillagavathie Pillay

Consultant Neonatologist, University Hospitals Leicester NHS Trust, Department of Neonatology

Professor of Neonatology, Research Institute for Health Related Sciences, Faculty of Science and Engineering, University of Wolverhampton

Professor Natalie Armstrong

Professor of Healthcare Improvement Research

Department of Health Sciences, University of Leicester, United Kingdom

For the OPTI-PREM Study Team

Keywords: neonatology, place of care, decision-making, qualitative research

Word count: 4273

For peer review only

## Abstract:

**Objectives:** Preterm babies born between 27 and 31 weeks of gestation in England are usually born and cared for in either a Neonatal Intensive Care Unit or a Local Neonatal Unit—with such units forming part of Operational Delivery Networks. As part of a national project seeking to optimise service delivery for this group of babies (OPTI-PREM), we undertook qualitative research to better understand how decisions about place of birth and care are made and operationalised.

**Design:** Qualitative analysis of ethnographic observation data in neonatal units and semi-structured interviews with neonatal staff.

**Setting:** Six neonatal units across two neonatal networks in England. Two were Neonatal Intensive Care units and four were Local Neonatal Units.

**Participants:** Clinical staff (n=15) working in neonatal units, and people present in neonatal units during periods of observation.

**Results:** In the context of real-world neonatal practice, with multiple (and rapidly-evolving) uncertainties relating to mothers, babies, and unit/network capacity, 'best place of care' protocols were only one element of much more complex decision-making processes. Staff often made judgements from a less-than-ideal starting point, and were forced to respond to evolving clinical and organisational factors. In particular, we report that managerial considerations relating to demand and capacity organised decision-making; demand and capacity management was time-consuming and generated various pressures on families, and tensions between staff.



For peer review only - http://bmjopen.bmj.com/site/about/guidelines.xhtml

**Conclusions:**

Researchers and policymakers should take account of the organisational context within which place of care decisions are made. The dominance of demand and capacity management considerations is likely to limit the impact of other improvement interventions, such as initiatives to integrate families into the neonatal care provision. Demand and capacity management is an important element of neonatal care that may be overlooked, but significantly organises how care is delivered.

## Strengths and limitations of this study

- This study is the first to use qualitative methods to explore practices relating to 'best place of care' using data from real-world neonatal practice.
- We undertook extensive observation (280hrs) in six neonatal care units (NICU and LNU), and captured the perspectives of a range of neonatal staff (doctors, nurses, transport specialists).
- We interviewed only a few staff from each unit, thus limiting the analytic 'depth' and comparison between sites.
- Our data did not include observations/interviews in obstetrics or maternity care, or with neonatal transport services, neonatal network managers or cot locating facilities, and we therefore do not discuss the operational arrangements and interdependencies between these services.



For peer review only - http://bmjopen.bmj.com/site/about/guidelines.xhtml

## INTRODUCTION

Babies needing medical care after birth are cared for in three types of neonatal unit across England, organised into Operational Delivery Networks: Neonatal Intensive Care Units (NICU), Local Neonatal Units (LNU), and Special Care Baby Units (SCBU). Evidence suggests that outcomes are better when extremely preterm babies ($\leq$26 weeks of gestation) are born in a maternity service attached to a NICU.[1,2] For the next most vulnerable group of babies (i.e. those born between 27 and 31 weeks of gestation [hereafter referred to as born at 27-31 weeks]), there is little evidence to guide decisions about their place of birth and care. More evidence is available for babies born >31 weeks.[3] Babies born at 27-31 weeks represent around 12% of all preterm births each year in England, use twice as many neonatal bed days/year compared to extremely preterm babies, and account for over a third of all neonatal care days nationally.[4]

In the absence of clear evidence to guide optimal place of care, babies born at 27-31 weeks are born and cared for across both NICU and LNU. A national project seeking to optimise service delivery for this group of babies, OPTI-PREM,[4] is investigating whether new recommendations should be developed for subgroups of babies within the 27-31 week range, based on mortality or morbidity analyses. [Detailed criteria regarding weights and multiple babies at the lower end of the gestational age have been modified since this study was carried out.[5]] In practice, some women (who are known to be at high risk of complicated pre-term delivery) may be transferred to a specialist unit, but many others continue to deliver in a maternity service linked to a LNU. Some of these may be transferred post-delivery to a NICU due to their degree of illness or prematurity.[5] These ex-utero



For peer review only - http://bmjopen.bmj.com/site/about/guidelines.xhtml

transfers are known to create particular stresses for babies and families[6–9] and should be avoided where possible.[10] In the past, transfers have been shown to be related to regional demand and capacity issues.[11–13] However, a high number of transfers have continued despite neonatal service reorganisation.[14] There is little recent evidence on how decisions about place of care are made in practice.

This article draws on qualitative work exploring neonatal care for babies born 27-31 weeks, undertaken as part of the OPTI-PREM study. Using observation in neonatal units and interviews with staff, we investigate how decisions about place of care are made and operationalised for this group of babies. We are aware of no previous ethnographic work addressing place of care decision-making in this clinical context.

## METHODS

We undertook an ethnographic study in two Neonatal Operational Delivery Networks in England. Within each network, a NICU and two attached LNU were included in the study (six neonatal units in total). From January to October 2018, observations and staff interviews were conducted in order to explore decision-making about place of care in a real-world context.[15–17] The wider study also captured experiences of parents but as parents were not directly involved in making decisions about babies' place of care, these data will be reported separately.



For peer review only - http://bmjopen.bmj.com/site/about/guidelines.xhtml

Sites were identified and invited to participate through the study principal investigator's professional network. Staff working in the units were introduced to the study by members of the OPTI-PREM project team. Written information on the project was provided. Observations were conducted by AP (an experienced qualitative researcher), and included ward rounds, daily clinical activities, bedside discussions with parents, transfer discussions and referrals, and transfers themselves. Observations were guided by an observation framework [supplementary file]. Verbal consent for observations was sought from all staff and parents in the unit prior to each period of observation. Anonymised field notes were made. AP proactively responded to sensitive situations. Every fieldwork day, AP checked-in with staff to identify any babies and families in a particularly challenging situation (e.g. palliative care), and did not disturb these families. In emergency situations, or when it seemed otherwise appropriate, AP withdrew from the bedside area.

As well as undertaking informal discussions with staff and parents during ethnographic data collection, AP also conducted formal interviews with staff. All doctors and nurses working at the units on observation days were approached for interview. Participation was voluntary. Fifteen staff agreed to be formally interviewed (many more contributed to the ethnographic element of the study, through being observed and via informal chats), and written consent was obtained. Most non-participation was due to challenges with scheduling around shifts. Interviews were semi-structured based on a topic guide [supplementary file] developed through literature review and discussions within the project team. Interviews were conducted by AP on site, lasted up to an hour and were audio-recorded, transcribed verbatim, and de-identified



For peer review only - http://bmjopen.bmj.com/site/about/guidelines.xhtml

Analysis was inductive and interpretative, with AP using the constant comparative method to undertake initial coding.[18] CC also carried out detailed reading of transcripts/field notes, and employed an analytic approach known as 'institutional ethnography'[19–22] to study place of care decision-making as part of socially-organised work processes.[23–26] NVivo software was used to organise and retrieve data.

For peer review only

**Patient and Public Involvement**

The OPTI-PREM project was supported by a Bliss[27] volunteer parent panel, which was involved in designing and overseeing the research.

## RESULTS

In total, 280 hours of observations were conducted and 15 members of staff were interviewed across the six units. A breakdown of professional roles is shown in Table 1.

| Site | Designation | Clinician Type | Number of participants (Total=15) |
|---|---|---|---|
| Site 1 | NICU | Doctor (Consultant) | 1 |
| Site 2 | NICU | Doctor<br>Nurse | 1<br>1 |
| Site 3 | LNU | Doctor (Registrar)<br>Nurse | 1<br>2 |
| Site 4 | LNU | Doctor (Consultant)<br>Nurse | 1<br>1 |
| Site 5 | LNU | Doctor (Consultant) | 1 |
| Site 6 | LNU | Doctor (Consultant)<br>Nurse | 1<br>2 |



For peer review only - http://bmjopen.bmj.com/site/about/guidelines.xhtml

| | | | |
|---|---|---|---|
| Working across sites | | Transport Nurse | 3 |

Table 1: Participant overview

The findings reported here are presented in two main themes. The first theme highlights the complex, multi-factorial context in which staff made decisions about place of care, and consequently the difficulties of producing and applying 'best place of care' protocols, even for *subgroups* of babies within the 27-31 week range. The second theme specifically focuses on how staff integrated 'managerial thinking' about demand and capacity management (within the network's contractual framework) into clinical decision-making about the 'best place of care' for each individual baby.

**'Best place of care' protocols within contextualised decision-making processes**

Place of birth and care was to a large extent governed by regional protocols, which determined what should happen in the case of an expected preterm birth at 27-31 weeks gestation.[5,28,29] For example, regional care pathways meant that LNU should only accept and care for babies born at ≥27/28 weeks, depending on the unit. For expectant preterm births in earlier gestational subgroups, the woman (with baby in utero) should be transferred to a maternity service attached to a NICU, assuming mother and fetus were considered adequately stable. Otherwise, the baby could be transferred post-delivery to a NICU for ongoing care if needed. Neonatal staff looked to such protocols (along with dynamic assessment of unit/network resources) to determine how to proceed.



For peer review only - http://bmjopen.bmj.com/site/about/guidelines.xhtml

*Majority of the time, it's quite a simple decision. For example, guidelines [say] we can't deliver, or we shouldn't manage anyone less than 27 weeks here […] And [we] have clear guidelines of how many nurses we need to run an ITU [intensive therapy unit], how much space you need and all that.*

*Doctor12, LNU*

Although, as indicated above, these decisions were often simple *in principle*, they involved *in practice* multiple uncertainties relating to both mother and baby's condition. Staff knew that, in an idealised scenario, the safest possible option would be for all mothers to have access to the specialist resources of a maternity service linked to a NICU, but they also recognised that this level of specialist care was unnecessary for some babies. The problem was that, although some information was known prior to delivery (and an informed guess made about a baby's subsequent condition), a baby's post-delivery condition was unpredictable:

*These are [a] very notorious group because [babies] can behave like an extreme premature, or they can just need a bit of support […]. It's very difficult to predict these babies.*

*Doctor12, LNU*

Pre-delivery, it was difficult to predict which babies could be safely managed on LNU, and decisions were reliant on local expertise. Staff considered this critical decision-making expertise to be lacking in some LNU:



For peer review only - http://bmjopen.bmj.com/site/about/guidelines.xhtml

*We can scan but we don't have the expertise to confidently say there's no bleeds going on, or the head's normal. So small things add up to not having good care or optimal care for the extreme premature baby. [For example], we are not funded, we've not got the personnel or the skills, because we don't deal with that so often.*

*Doctor12, LNU*

Staff therefore understood that there was extra risk attached to delivering this group of babies in many LNU [expertise and equipment varied from unit to unit].

Not only were babies unpredictable, but so too were mothers and families. For example, a mother might not travel to the preferred NICU due to imminent labour, or social circumstances. Units therefore had to handle imminent deliveries regardless of whether or not they were equipped to care for the baby according to recognised standards and guidelines for neonatal care. Even mothers classified in advance as being 'high risk' (and therefore booked at a NICU) often presented in labour at their local unit, which was then obliged to manage the delivery:

*Triplets were booked for Site 1 but were born at Site 5. Consultant said this happens a lot—that high risk pregnancies are booked at Site 1 but then Mum presents at Site 5 too far along to be moved.*

*Observation, Site 5, LNU*



For peer review only - http://bmjopen.bmj.com/site/about/guidelines.xhtml

For peer review only

Often, a suboptimal delivery at an LNU was not due to the unpredictability of mother or baby, but due to capacity issues in local NICU or lack of availability of transport teams (that were needed to safely carry out some in-utero transfers). Units often ended up accepting babies for which they did not have appropriate capacity (e.g. equipment/staffing):

> *[The nurse said:] Sometimes [the LNU] is full and [they] tell people to rock up [arrive late, without warning] without checking [we] can take the pressure.*
>
> *Observation, Site 5, LNU*

A unit's own capacity management had to be undertaken in relation to capacity issues across the network/region. As the excerpt above indicates, units were not always aware of/responsive to other units' management concerns and this was exacerbated by variation in policies and fluctuating capacity across units of the same designation:

> *[…] every hospital is different. Policies are different. Maybe it will be in time standardised within the NHS*
>
> *Nurse10, NICU*

As highlighted above, many of the 27-31 week babies in this study were born in LNU—and this included those who did not meet established local criteria. [See Edwards and Impey[30] who found that, even in the <27 week group of extremely preterm babies, only 50% were actually born in a specialist unit, despite this being the recommendation.] In practice, protocols were frequently contravened due to limited capacity across obstetrics/maternity services and NICU, along with the other factors relating to mother and baby that we have



For peer review only - http://bmjopen.bmj.com/site/about/guidelines.xhtml

For peer review only

already outlined. Other babies were born in NICU (e.g. due to the mother's home being in close proximity) even though they could be adequately cared for on an LNU.

In practice, therefore, many place of care decisions were not taken from a stable, pre-clinical position, but within an existing clinical context (either LNU or NICU)—in which care for mother and baby was already underway and rapidly evolving:

> *If the birth is imminent then we just go for it. Personally I've dealt with babies who are twenty-three-and-a-half weeks onwards [at another unit]. So personally speaking I'm comfortable, and I'm pretty sure my nursing colleagues are pretty comfortable. So we deal with them, but as soon as the baby comes out, one of my colleagues starts looking around hunting for a bed.*
>
> *Doctor20, LNU*

In such situations, staff had to cope with whatever happened, and prepare to transfer the baby (if required) after delivery.

In other circumstances, staff made calculated judgements to keep mothers at a local hospital, based on their experience and dynamic knowledge of multiple contextual factors:

> *On a December cold, wintery night and a mum came in-utero [with triplets]. And there was possibly a small window for her to go elsewhere, but I carefully calculated the risk and said "your twenty-nine-weekers have very good growth for all those babies and, it's winter and there is a chance that things might happen en route...". At the time I had a very experienced registrar with me*



For peer review only - http://bmjopen.bmj.com/site/about/guidelines.xhtml

For peer review only

*and I did have another member of the team who could definitely come back [into work after leaving shift]. And this mum had had steroids as well in the last week. So looking at all those calculations I actually did look after them.*

*Doctor30, LNU*

Assessment of the best place of care was often re-evaluated following delivery, with some decisions being relatively straightforward (in principle at least)—based on urgent clinical needs—whilst others required more complex assessment of the pros and cons within the wider unit/network context.

When staff made decisions (with baby either in-utero or ex-utero), they not only applied protocols but also attended to a huge range of uncertainties and a rapidly-evolving situation relating to mother, baby, and also unit/network capacity, which affected how those protocols were applied. Family circumstances and preferences were sometimes accommodated in decision-making (e.g. when mother and baby were both inpatients, efforts were made to locate them at the same hospital), but decisions were largely determined by the clinical needs of the baby, organised within a wider demand and capacity management framework—as discussed below.

**'Thinking managerially' about where individual babies should be born and cared for**

Decisions about place of care for most of the babies born at 27-31 weeks was highly contextualised and involved assessment of the particular needs of individual babies. It was clear that all staff aspired to the best quality care for individual babies. However, each individual baby's clinical assessment was made within a socially-organised clinical context.



For peer review only - http://bmjopen.bmj.com/site/about/guidelines.xhtml

Our data show that resources (and their distribution across networks) significantly shaped place of care decisions about individual babies born at 27-31 weeks—with specialist maternity services and NICU only being available in city locations, and without the necessary capacity to accommodate all 27-31 week births.

More specifically, we found that a large amount of staff work was dedicated to matching the (either anticipated or known) clinical needs of the neonatal baby population ('demand') with the availability of expertise and technologies ('capacity')—both of which were scattered geographically across a region, and were constantly being reconfigured. Staff drew not only on knowledge of the baby's needs (e.g. requirement for ventilation, tube feeding, surgery), but also on knowledge of capacity and resources within their own unit within the context of the wider network:

> *We like to think managerially as well [as clinically]. If [another unit with limited specialist capacity] has a twenty-eight week [baby] and they ask whether we would like to accept, we are more than happy. Because, not only it's good for our practice that we keep on getting prem babies, it [also] brings money to the [hospital]. So we are always looking for kids…*
>
> *Doctor20, LNU*

Staff spoke of their 'network responsibility' to try to accommodate babies—a responsibility linked to national tariffs for different categories of care[31,32] and locally negotiated contracts and payments.[33] They knew that, in relation to this 'responsibility', they needed to "think managerially" about capacity and resources. Such thinking (and the associated decision-making) was concerned with the unit's need to maximise income, whilst avoiding



For peer review only - http://bmjopen.bmj.com/site/about/guidelines.xhtml
For peer review only

‘unsafe’ practice (as determined by capacity/staffing protocols[10]). In the excerpt above, one unit was short of the required capacity, whilst another was looking to ensure that available capacity was generating income.

Staff were locked into continuous work to review their own capacity and respond to the requests of other units within the network. ‘Capacity’ in this context included many different elements—including staff mix, qualifications, experience, space, and technologies:

> *So in our [LNU], [we can take] twenty eight weeks and above. And then we look at ourselves. How are we? Is there enough nursing staff? Are there enough doctors? And then we look at bed capacity, and [intensive care capacity], whether there’s enough space. […] Sometimes it happens that we shift babies out because of capacity.*
>
> *Doctor20, LNU*

All units logged requests for transfers from other units, noting why they accepted or refused. One transport nurse emphasised that units did their best to accommodate babies who were vulnerable to deterioration as a consequence of transfer:

> *“[Units] try and shuffle to make [babies] fit in.”*
>
> *TransportNurse05A*

However, in all six units, staff or cot capacity were the most often cited reasons for a refusal.



For peer review only - http://bmjopen.bmj.com/site/about/guidelines.xhtml

Units were paid for their 'activity' (number of babies), so accepting new babies was important for income generation as well as to maintain the unit's status (also linked to the category of babies they were allowed to accept):

> *You have to accept, because we are an LNU, we cannot afford our activity to go down. Because [then] we'll be relegated to a [non-specialist] unit. So we won't want to be seen as refusing [...] We can get de-skilled [...] and the more activity we do, the more funds we get [so] it's a win-win situation.*
>
> *Doctor26, LNU*

LNU therefore tried to retrieve babies who had been transferred to NICU, as soon as they were clinically ready for repatriation:

> *There is a baby here from an out-of-network hospital. [The hospital] has been ringing to see if it can come back.*
>
> *Observation, Site 1, NICU*

In addition, each baby attracted a different tariff, according to the category of care that was required[31,32]—and this was an important consideration:

> *While waiting, [another hospital] has rung up with an ex-utero transfer (twins). "We must be the only place with beds!" Everyone is excited to take the babies as it is an ITU transfer and that brings in a lot of money. [Staff member] singing "one thousand and twenty-one pounds. Thank you!"*
>
> *Observation, Site 3, LNU*



For peer review only - http://bmjopen.bmj.com/site/about/guidelines.xhtml

However, maximising capacity, and meeting the needs of very sick babies, was sometimes in tension with providing a broad spectrum of neonatal delivery for the local population. For example, babies who were clinically stable, and whose local unit was a NICU, were often transferred out, sometimes to an inconvenient location, in order to accommodate babies with more complex needs:

> *The units are very good; if it is a really sick baby that's got a specific problem then they're very good at trying to make beds, so often we'll move other babies out … we do jiggling around quite a lot.*
>
> *TransportNurse05*

> *So it's finding a divide between running a hospital (just purely thinking about the service and how much activity you can churn through at the high end of intensive care) versus running a service that makes sure you provide the right service for the families of your local population.*
>
> *Doctor06, NICU*

Unlike decisions that were made based purely on clinical need, decisions orientated entirely to demand and capacity management took an emotional toll on staff:

> *I think the hardest conversation is the move out for capacity reasons. So making a decision to impact significantly on a family […] for the care of a different baby who they don't have nothing to do with can be quite difficult sometimes.*
>
> *Doctor09, NICU*

Capacity-related issues could also create tension between staff groups:



For peer review only - http://bmjopen.bmj.com/site/about/guidelines.xhtml

*The doctor was complaining about how there is no standardised practice for closing the unit […] The nurses always want to operate at a gold standard, but it is impossible to operate at that level because the unit is always falling short due to staff shortages.*

*Observation, Site 1, NICU*

Senior staff were drawn into extensive work to account for capacity-related decisions, and they passed this pressure onto staff within their units:

*Consultants want to have a better way of recording why babies can't be accepted [for upwards reporting]. They want specifics, so they were saying things like "Why have you said we're busy? What is it about the category of babies in today that means we can't take another one?"*

*Observation, Site 1, NICU*

In practice, 'clinical' and 'managerial' ways of thinking were entirely intertwined within the work of neonatal staff, including their decision-making about place of care.

## DISCUSSION

This paper provides empirical evidence of how 'best place of care' protocols are incorporated into practical, real-world decision-making for babies born between 27-31 weeks of gestation in England. Our findings highlight that clinical decisions about the 'best place of care' for individual babies are, in practice, and to a significant extent, orientated to demand and capacity management, as part of a unit's contractual arrangements within the network. We have drawn attention to the managerial work that is involved in staff decision-



For peer review only - http://bmjopen.bmj.com/site/about/guidelines.xhtml

making about place of care. When staff make these decisions for an individual baby, they integrate clinical knowledge about that baby's needs with management understandings of what is 'best'. We have highlighted that neonatal staff are highly skilled at making complex judgements about place of care based on their in-situ knowledge about the needs of babies, and the resources (e.g. staffing, equipment, cots) that are available to meet those needs.

Although sometimes it appears that 'clinical' and 'managerial' ways of knowing are aligned (and appear to present no difficulties for staff, parents, or babies), at other times they come into tension. Institutional systems and processes relating to demand and capacity management (contractual funding arrangements, safe staffing protocols etc.) often create difficulties as they coordinate neonatal work. Overall, a large amount of staffing resource is involved in negotiating between babies' individual needs and units' resources. Staff spend considerable energies balancing the competing needs of babies (whether they are transferred or stay put in their birth hospital), within the constantly-reconfiguring distribution of resources within a network. This demand and capacity management work inevitably diverts staff from other activities, and impacts morale, as well as their time to support babies and families. This is likely to have a negative impact on important partnership-working with parents.[34] Parents may also be faced with considerable knock-on challenges in order to maintain their active parenting role, as demand and capacity considerations take precedence and mother and baby are 'juggled' between units. Our findings align with studies in Canada, which have made visible the considerable parental work involved in neonatal care (e.g. in transfers, Family Integrated Care) and, importantly, have highlighted dissonance with major protocol-driven institutional systems, which fail to



For peer review only - http://bmjopen.bmj.com/site/about/guidelines.xhtml

take account of this work.[9,35] Our study contributes to a small corpus of work which can support stakeholders understandings of how contractual and funding configurations shape clinical decision-making (and impact on care) in practice.[36] This institutional context, and analysis of the practical activities involved, have frequently been absent from research on neonatal place of care and transfers.

Our findings are highly relevant to stakeholders who see solutions in standardised protocols and pathways to deliver improvements. We found that place of care protocols were only one element within much more complex decision-making processes. This observation has implications for both the production and application of evidence relating to 'best place of care'. In relation to *production* of evidence, studies attempting to assess the clinical outcomes of babies born on NICU compared with those born on LNU are likely to be challenging as NICU and LNU vary considerably within these classifications, and the characteristics of babies born in each are not standardised. Transfers between units are also likely to make comparisons extremely difficult. In relation to *application* of evidence, standardised protocols for this group of babies may also present difficulties within a context that involves un-standardised babies, mothers, geographies, resourcing and so on. Even if future evidence indicates that some subgroups of babies in the 27-31 week range are (theoretically) best born and/or cared for in hospitals with a NICU, such highly specialist and costly facilities will not be on the doorstep of all mothers, and will undoubtedly have limited capacity. Stakeholders should be alert to the situatedness of decision-making and the potential for unintended consequences following the introduction of new protocols.[37]



For peer review only - http://bmjopen.bmj.com/site/about/guidelines.xhtml

There are some limitations to this study. Data are based on a sample of neonatal units that were purposively selected for the project. Within units, data collection did not include observations or interviews in obstetrics or maternity care, or with neonatal transport services, neonatal network managers or cot locating facilities, and we therefore do not discuss the operational arrangements and interdependencies between these services and neonatal units. Future work might look in more detail at this intersection. Additionally, only a few staff from each unit were interviewed formally, thus limiting the analytic 'depth' and comparison between sites. Nevertheless, our findings provide important insights into the real-world context in which place of care decisions are made and operationalised.

## CONCLUSIONS

Researchers and policymakers should take account of the organisational context in which place of care decisions for pre-term babies (e.g. born 27-31 weeks) are made. Although some standardised guidelines are likely to be useful, it will continue to be necessary for staff to make on-the-spot, skilled judgements about place of care. It is important that this skilled work is recognised in workforce and wider neonatal care planning. Notably, this study found that place of care decisions are significantly orientated to demand and capacity management—alongside other, more obvious, clinical considerations. In practice, the dominance of these management considerations is likely to limit the impact of other improvement interventions, such as initiatives to integrate families into the neonatal care provision. Demand and capacity management is an important element of neonatal care that may be overlooked, but significantly organises how care is delivered.



For peer review only - http://bmjopen.bmj.com/site/about/guidelines.xhtml

## Acknowledgements

The authors thank all the staff whose experiences are reported in this article, and the other participants involved in the OPTI-PREM study. We are also grateful to the reviewers, especially Catherine Ringham, for their helpful comments.

Natalie Armstrong is supported by a Health Foundation Improvement Science Fellowship and also by the National Institute for Health Research (NIHR) Applied Research Collaboration East Midlands (ARC EM). The views expressed are those of the authors and not necessarily those of the NHS, the NIHR or the Department of Health and Social Care.

The OPTI-PREM study team: Natalie Armstrong; Victor L Banda; Vasiliki Bountziouka; Caroline Cupit; Kelvin Dawson; Elaine M Boyle; Elizabeth S Draper; Abdul Qader T Ismail; Bradley Manktelow; Neena Modi; Alexis Paton; Oliver Rivero-Arias; Sarah E Seaton; Miaoqing Yang; and Thillagavathie Pillay (Chief Investigator).

## Author contributions

Obtained funding: TP, EB, NA. Study design: NA. Data analysis: CC, AP. Drafted manuscript: CC, AP. Interpreted to neonatal practice: TP, EB. Conceptualised and critically revised the manuscript: CC. All authors approved the final version.

CC and AP contributed equally to this paper.



For peer review only - http://bmjopen.bmj.com/site/about/guidelines.xhtml

## Funding

This work was supported by the National Institute for Health Research, Health Services and Delivery Research Stream, Project number 15/70/104.

## Competing interests

None declared.

## Disclaimer

The views expressed in this article are those of the authors and not necessarily those of the NHS or NIHR.

## Patient consent for publication

Not required

## Ethics approval

The study was approved by the North East – Tyne and Wear South REC (IRAS 212304) and is registered with Clinicaltrials.gov (NCT02994849).

## Provenance and peer review

Not commissioned. Externally peer-reviewed.

## Data availability statement

No data are available. The consent for this study restricts use of transcripts to the study team for confidentiality reasons.

For peer review only

## References

1 Watson SI, Arulampalam W, Petrou S, *et al.* The effects of designation and volume of neonatal care on mortality and morbidity outcomes of very preterm infants in England: retrospective population-based cohort study. *BMJ Open* 2014;**4**:e004856. doi:10.1136/bmjopen-2014-004856

2 Helenius K, Longford N, Lehtonen L, *et al.* Association of early postnatal transfer and birth outside a tertiary hospital with mortality and severe brain injury in extremely preterm infants: observational cohort study with propensity score matching. *BMJ* 2019;**367**:l5678. doi:10.1136/bmj.l5678

3 Boyle EM, Johnson S, Manktelow B, *et al.* Neonatal outcomes and delivery of care for infants born late preterm or moderately preterm: a prospective population-based study. *Arch Dis Child - Fetal Neonatal Ed* 2015;**100**:F479–85. doi:10.1136/archdischild-2014-307347

4 Pillay T, Modi N, Rivero-Arias O, *et al.* Optimising neonatal service provision for preterm babies born between 27 and 31 weeks gestation in England (OPTI-PREM), using national data, qualitative research and economic analysis: a study protocol. *BMJ Open* 2019;**9**. doi:10.1136/bmjopen-2019-029421

5 NHS England. Implementing the Recommendations of the Neonatal Critical Care Transformation Review. 2019. https://www.england.nhs.uk/publication/implementing-the-recommendations-of-the-neonatal-critical-care-transformation-review/ (accessed 28 Oct 2021).

6 Sommer CM, Cook CM. Disrupted bonds–parental perceptions of regionalised transfer of very preterm infants: a small-scale study. *Contemp Nurse* 2015;**50**:256–66.

7 Ballantyne M, Orava T, Bernardo S, *et al.* Parents' early healthcare transition experiences with preterm and acutely ill infants: a scoping review. *Child Care Health Dev* 2017;**43**:783–96. doi:10.1111/cch.12458

8 Aagaard H, Hall EOC, Ludvigsen MS, *et al.* Parents' experiences of neonatal transfer. A meta-study of qualitative research 2000–2017. *Nurs Inq* 2018;**25**:e12231. doi:10.1111/nin.12231

9 Ringham CL, MacKinnon K. Mothering Work and Perinatal Transfer: An Institutional Ethnographic Investigation. *Can J Nurs Res* 2019;:0844562119884388.

10 British Association of Perinatal Medicine. Optimal Arrangements for Neonatal Intensive Care Units in the UK including guidance on their Medical Staffing A Framework for Practice. London: 2021. https://www.swneonatalnetwork.co.uk/media/182167/bapm_optimal_arrangement_for_nicus_revision_21-4-21.pdf (accessed 25 Oct 2021).

11 Fenton AC, Leslie A, Skeoch CH. Optimising neonatal transfer. *Arch Dis Child - Fetal Neonatal Ed* 2004;**89**:F215–9. doi:10.1136/adc.2002.019711



For peer review only - http://bmjopen.bmj.com/site/about/guidelines.xhtml

12 Gill AB, Bottomley L, Chatfield S, *et al.* Perinatal transport: problems in neonatal intensive care capacity. *Arch Dis Child - Fetal Neonatal Ed* 2004;**89**:F220–3. doi:10.1136/adc.2003.028159

13 Fowlie PW, Booth P, Skeoch CH. Moving the preterm infant. *BMJ* 2004;**329**:904–6. doi:10.1136/bmj.329.7471.904

14 Gale C, Santhakumaran S, Nagarajan S, *et al.* Impact of managed clinical networks on NHS specialist neonatal services in England: population based study. *BMJ* 2012;**344**:e2105. doi:10.1136/bmj.e2105

15 Savage J. Ethnography and health care. *BMJ* 2000;**321**:1400–2.

16 Dixon-Woods M. What can ethnography do for quality and safety in health care? *Qual Saf Health Care* 2003;**12**:326–7.

17 Leslie M, Paradis E, Gropper MA, *et al.* Applying ethnography to the study of context in healthcare quality and safety. *BMJ Qual Saf* 2014;**23**:99–105. doi:10.1136/bmjqs-2013-002335

18 Glaser BG, Strauss AL. *The Discovery of Grounded Theory: Strategies for Qualitative Research*. Aldine Publishing Company 1967.

19 Smith DE. *The Everyday World as Problematic: A Feminist Sociology*. Toronto: : University of Toronto Press 1987.

20 Campbell M, Gregor FM. *Mapping Social Relations: A Primer in Doing Institutional Ethnography*. Toronto: : University of Toronto Press 2002.

21 Smith DE. *Institutional Ethnography as Practice*. Washington DC: : Rowman & Littlefield 2006.

22 Rankin J. Conducting Analysis in Institutional Ethnography: Guidance and Cautions. *Int J Qual Methods* 2017;**16**:1609406917734472. doi:10.1177/1609406917734472

23 Sinding C, Hudak P, Wiernikowski J, *et al.* "I like to be an informed person but…" negotiating responsibility for treatment decisions in cancer care. *Soc Sci Med* 2010;**71**:1094–101. doi:10.1016/j.socscimed.2010.06.005

24 Campbell ML, Rankin JM. Nurses and electronic health records in a Canadian hospital: examining the social organisation and programmed use of digitised nursing knowledge. *Sociol Health Illn* 2016;**39**:365–79. doi:10.1111/1467-9566.12489

25 Reid L, Kydd A, Slade B. An inquiry into what organised difficult advance care planning conversations in a Scottish residential care home using institutional ethnography. *J Res Nurs* 2018;**23**:220–36. doi:10.1177/1744987118756477

26 Cupit C, Rankin J, Armstrong N, *et al.* Overruling uncertainty about preventative medications: the social organisation of healthcare professionals' knowledge and practices. *Sociol Health Illn* 2020;**42**:114–29. doi:10.1111/1467-9566.12998



For peer review only - http://bmjopen.bmj.com/site/about/guidelines.xhtml

27 Bliss. What we do. 2020.https://www.bliss.org.uk/about-us/what-we-do (accessed 16 Mar 2020).

28 British Association for Perinatal Medicine. Optimal Arrangements for Neonatal Intensive Care Units in the UK. British Association for Perinatal Medicine 2014. https://www.bapm.org/resources/31-optimal-arrangements-for-neonatal-intensive-care-units-in-the-uk-2014 (accessed 25 Nov 2019).

29 British Association of Perinatal Medicine. Neonatal Service Quality Indicators: Standards relating to structures and processes. British Association of Perinatal Medicine 2017. https://www.bapm.org/resources/11-neonatal-service-quality-indicators-standards-relating-to-structures-and-processes-2017 (accessed 25 Nov 2019).

30 Edwards K, Impey L. Extreme preterm birth in the right place: a quality improvement project. *Arch Dis Child - Fetal Neonatal Ed* Published Online First: 12 November 2019. doi:10.1136/archdischild-2019-317741

31 BAPM. Categories of care 2011. 2011.https://www.sort.nhs.uk/Media/SONeT/Guidelines/Nationalpolicies/BAPM%202011%20categories%20of%20care.pdf

32 NHS England. National Programmes of Care and Clinical Reference Groups: Neonatal Critical Care Service Specification. 2016. https://www.england.nhs.uk/commissioning/spec-services/npc-crg/group-e/e08/ (accessed 4 Feb 2020).

33 NHS England. Implementing the Recommendations of the Neonatal Critical Care Transformation Review. 2016. https://www.england.nhs.uk/publication/implementing-the-recommendations-of-the-neonatal-critical-care-transformation-review/ (accessed 4 Feb 2020).

34 Brødsgaard A, Pedersen JT, Larsen P, *et al.* Parents' and nurses' experiences of partnership in neonatal intensive care units: A qualitative review and meta-synthesis. *J Clin Nurs* 2019;**28**:3117–39. doi:10.1111/jocn.14920

35 Ringham C, Rankin J, Marcellus L. The Social Organization of Neonatal Nurses' Feeding Work. *Neonatal Netw* 2020;**39**:283–92. doi:10.1891/0730-0832.39.5.283

36 Griffith AI, Smith DE, editors. *Under New Public Management: Institutional Ethnographies of Changing Front-line Work*. Toronto: : University of Toronto Press 2014.

37 Timmermans S, Epstein S. A world of standards but not a standard world: toward a sociology of standards and standardization. *Annu Rev Sociol* 2010;**36**:69–89.



For peer review only - http://bmjopen.bmj.com/site/about/guidelines.xhtml

**OPTI-PREM Interview Schedule – Staff**

*N.B. This topic guide will be used flexibly – the interviewer will be responsive to each individual participant and will explore related issues of interest as they arise.*

**Welcome and introduction**

- Confirm that the interview is solely for the use of the researchers and any discussions will not be communicated in any way to anyone else.
- The report will pull together findings from all participants in the study and no individual will be identified.
- We like to audio-record each interview – is that ok?

**Reiterate aims of interview**

- To understand staff's views and experiences and make sure these are included in discussions about the optimal place of care for babies born between 27 and 31 weeks of gestation.
- We are interested in your views and experiences based on the type of work you do. If you do not feel you are able to comment on any area please say so.
- Do you have any questions before we start?

**Background to participant**

- Please could you tell me a little bit about yourself?
  - Prompt for: role, experience, seniority

**Approach to decision-making on optimal place of care for these babies**

- What informs decision-making on optimal place of care for these babies?
- Who is involved?
  - How/why?
- What else influences this?
  - Prompt for: baby, parents, guidelines/protocols, cot capacity
- How easy or difficult is it to decide on the optimal place of care for this type of baby?

**Transfers in place of care**

- How do you go about exploring or bringing about a transfer?
- What are some of the things that help with this?
- What are some of the things that make this difficult?

**Relationship with parents**

- How do you seek to work with parents about place of care and any transfers?
  - Why is this your approach?
  - Has it always been this way?
- Do you always manage to achieve this?
  - How/why?
- What are some of the challenges here?
- What makes this easier?

**Importance of place of care**

- To what extent do you think place of care is important for this group of babies?
- How much do you know about whether and how this might affect their outcomes?
- Do you have concerns about babies in sub-optimal care locations?
  - Babies in too low a level unit

For peer review only

- Babies in too high a level unit

**Impact on staff**

- Tell me about some of the ways seeking to optimise place of care for these babies impacts on you and your work.
- Thinking particularly about decisions about and any changes in a baby's place of care, what works well for you?
    - Prompt for more detail on why/how
- What could be better?
    - Why is this a problem?
    - How could it be improved?

**Anything not covered?**

- Is there anything that we haven't covered in the interview that you think we should know or think about for this project?

**Closing and thanks**

- Check that the participant is still happy for us to use all the information provided and offer the possibility to erase sections of the recording.
- Thank for their time and contribution.

For peer review only

**OPTI-PREM observation framework**

The observer will take written notes during the observations within neonatal units. Key issues to be observed are as follows, but additional relevant information should also be recorded:

- What is the event or type of activity being observed?
- What context and setting are you in?
- How does optimal place of care feature in what you're observing?
- What role does/will it play?

- Who is involved and how is the interaction managed?
- How, when and why is optimal place of care made relevant to what you are observing?
- How is optimal place of care positioned relative to other relevant issues (e.g. unit capacity, parental experience)?

- How is the idea of optimal place of care introduced to parents, and how is it received?
- Is there broad agreement between parent and professional, or are there tensions?

- How are decisions made about optimal place of care?
- What factors influence this?
- What will happen after the episode you are observing?

- How are transfers of place of care managed?
- What factors facilitate this?
- What factors make this more difficult?

Version 1 29/08/17
IRAS 212034

For peer review only

## Consolidated criteria for reporting qualitative studies (COREQ): 32-item checklist

Please indicate in which section each item has been reported in your manuscript. If you do not feel an item applies to your manuscript, please enter N/A.

For further information about the COREQ guidelines, please see Tong *et al.*, 2017: https://doi.org/10.1093/intqhc/mzm042

| No. | Item | Description | Section # |
|---|---|---|---|
| **Domain 1: Research team and reflexivity** | | | |
| Personal characteristics | | | |
| *1.* | Interviewer/facilitator | Which author/s conducted the interview or focus group? | |
| *2.* | Credentials | What were the researcher's credentials? *E.g. PhD, MD* | |
| *3.* | Occupation | What was their occupation at the time of the study? | |
| *4.* | Gender | Was the researcher male or female? | |
| *5.* | Experience and training | What experience or training did the researcher have? | |
| Relationship with participants | | | |
| *6.* | Relationship established | Was a relationship established prior to study commencement? | |
| *7.* | Participant knowledge of the interviewer | What did the participants know about the researcher? *E.g. Personal goals, reasons for doing the research* | |
| *8.* | Interviewer characteristics | What characteristics were reported about the interviewer/facilitator? *E.g. Bias, assumptions, reasons and interests in the research topic* | |
| **Domain 2: Study design** | | | |
| Theoretical framework | | | |
| *9.* | Methodological orientation and theory | What methodological orientation was stated to underpin the study? *E.g. grounded theory, discourse analysis, ethnography, phenomenology, content analysis* | |
| Participant selection | | | |
| *10.* | Sampling | How were participants selected? *E.g. purposive, convenience, consecutive, snowball* | |
| *11.* | Method of approach | How were participants approached? *E.g. face-to-face, telephone, mail, email* | |
| *12.* | Sample size | How many participants were in the study? | |
| *13.* | Non-participation | How many people refused to participate or dropped out? What were the reasons for this? | |
| Setting | | | |
| *14.* | Setting of data collection | Where was the data collected? *E.g. home, clinic, workplace* | |
| *15.* | Presence of non-participants | Was anyone else present besides the participants and researchers? | |

For peer review only

| | | | |
|---|---|---|---|
| *16.* | Description of sample | What are the important characteristics of the sample? *E.g. demographic data, date* | |
| Data collection | | | |
| *17.* | Interview guide | Were questions, prompts, guides provided by the authors? Was it pilot tested? | |
| *18.* | Repeat interviews | Were repeat interviews carried out? If yes, how many? | |
| *19.* | Audio/visual recording | Did the research use audio or visual recording to collect the data? | |
| *20.* | Field notes | Were field notes made during and/or after the interview or focus group? | |
| *21.* | Duration | What was the duration of the interviews or focus group? | |
| *22.* | Data saturation | Was data saturation discussed? | |
| *23.* | Transcripts returned | Were transcripts returned to participants for comment and/or correction? | |
| **Domain 3: analysis and findings** | | | |
| Data analysis | | | |
| *24.* | Number of data coders | How many data coders coded the data? | |
| *25.* | Description of the coding tree | Did authors provide a description of the coding tree? | |
| *26.* | Derivation of themes | Were themes identified in advance or derived from the data? | |
| *27.* | Software | What software, if applicable, was used to manage the data? | |
| *28.* | Participant checking | Did participants provide feedback on the findings? | |
| Reporting | | | |
| *29.* | Quotations presented | Were participant quotations presented to illustrate the themes / findings? Was each quotation identified? *E.g. Participant number* | |
| *30.* | Data and findings consistent | Was there consistency between the data presented and the findings? | |
| *31.* | Clarity of major themes | Were major themes clearly presented in the findings? | |
| *32.* | Clarity of minor themes | Is there a description of diverse cases or discussion of minor themes? | |

**When submitting your manuscript via the online submission form, please upload the completed checklist as a Figure/supplementary file.**

**If you would like this checklist to be included alongside your article, we ask that you upload the completed checklist to an online repository and include the guideline type, name of the repository, DOI and license in the *Data availability* section of your manuscript.**

Developed from: Allison Tong, Peter Sainsbury, Jonathan Craig, Consolidated criteria for reporting qualitative research (COREQ): a 32-item checklist for interviews and focus groups, International Journal for Quality in Health Care, Volume 19, Issue 6, December 2007, Pages 349–357, https://doi.org/10.1093/intqhc/mzm042
